# Self-assembling dipeptide antibacterial nanostructures with membrane disrupting activity

**DOI:** 10.1038/s41467-017-01447-x

**Published:** 2017-11-08

**Authors:** Lee Schnaider, Sayanti Brahmachari, Nathan W. Schmidt, Bruk Mensa, Shira Shaham-Niv, Darya Bychenko, Lihi Adler-Abramovich, Linda J. W. Shimon, Sofiya Kolusheva, William F. DeGrado, Ehud Gazit

**Affiliations:** 1 Department of Molecular Microbiology and Biotechnology, George S. Wise Faculty of Life Sciences, Tel Aviv University, Tel Aviv, 69978 Israel; 2 Department of Pharmaceutical Chemistry, Cardiovascular Research Institute, University of California, San Francisco, CA 94158 USA; 3 Department of Oral Biology, The Goldschleger School of Dental Medicine, Sackler Faculty of Medicine, Tel Aviv University, Tel Aviv, 69978 Israel; 4 Department of Chemical Research Support, Weizmann Institute of Science, Rehovot, 76100 Israel; 5 Ilse Katz Institute for Nanotechnology, Ben Gurion University of the Negev, Beer Sheva, 84105 Israel; 6 Department of Materials Science and Engineering, Iby and Aladar Fleischman Faculty of Engineering, Tel Aviv University, Tel Aviv, 69978 Israel

## Abstract

Peptide-based supramolecular assemblies are a promising class of nanomaterials with important biomedical applications, specifically in drug delivery and tissue regeneration. However, the intrinsic antibacterial capabilities of these assemblies have been largely overlooked. The recent identification of common characteristics shared by antibacterial and self-assembling peptides provides a paradigm shift towards development of antibacterial agents. Here we present the antibacterial activity of self-assembled diphenylalanine, which emerges as the minimal model for antibacterial supramolecular polymers. The diphenylalanine nano-assemblies completely inhibit bacterial growth, trigger upregulation of stress-response regulons, induce substantial disruption to bacterial morphology, and cause membrane permeation and depolarization. We demonstrate the specificity of these membrane interactions and the development of antibacterial materials by integration of the peptide assemblies into tissue scaffolds. This study provides important insights into the significance of the interplay between self-assembly and antimicrobial activity and establishes innovative design principles toward the development of antimicrobial agents and materials.

## Abstract (web summary)

Peptide-based supramolecular assemblies are a promising class of nanomaterials with important biomedical applications, but their antibacterial properties can be overlooked. Here the authors show the antibacterial activity of self-assembled diphenylalanine, which emerges as the minimal model for antibacterial supramolecular polymers.

## Introduction

Self-assembling molecules, including peptides, proteins, lipids, and nucleic acids, are the central building blocks of life that allow for the formation of cell membranes, cytoskeletal structures, and the extracellular matrix^1,2^. Extensive studies and mechanistic insights into the biophysical properties of these assemblies have allowed for the harnessing of the self-assembly process for various nano- and bio-technological applications^2–5^. For biomedical purposes, the development of self-assembled peptide nano-materials has primarily focused on the fields of regenerative medicine and drug delivery due mainly to the advantageous characteristics of these materials such as high biocompatibility levels, chemical diversity, high loading capacity, and extended circulation^6–10^. However, few studies have been carried out on the actual intrinsic antibacterial activity of peptide-based nanostructures, with the development of self-assembling materials with antibacterial capabilities primarily concentrated on complex non-peptide-based polymers and various biomimetics, and those based on peptide assemblies have been focused on relatively long and mainly hydrogel forming moieties^6,7^.

Peptide self-assembly is naturally utilized for fighting infections. Organisms from all kingdoms of life, including humans, produce potent membrane-targeting antimicrobial peptides as part of their innate immune response^11–13^. Recent reports have shown that a growing number of these peptides, including dermaspetin S9, protegrin-1, and human α-defensin 6, among many others^14–23^, as well as bacterially secreted peptides^24^, are able to self-assemble and form fibrillar amyloid-like nanostructures that play a role in their innate immune activity. Furthermore, additional members of the immune system, such as the mitochondrial antiviral signaling protein (MAVS), have been shown to undergo conformational changes and form amyloid-like structures that enable them to carry out their activity^25^. These structures closely resemble classical amyloidogenic assemblies, which are exceptionally stable unbranched, fibrillar, cross β-sheet quaternary protein, and polypeptide structures^26,27^ that attain their stability through noncovalent interactions, including hydrogen bonds, hydrophobic interactions, and π–π stacking interactions^28^. Interestingly, amphipathic antimicrobial peptides and short amyloid-related peptides share a similar high frequency of aromatic amino acids that have been suggested to play a role in the acceleration and stabilization of the amyloidogenic assemblies^28–30^. Moreover, classical amyloid proteins and peptides, such as serum amyloid A, β-amyloid, α-synuclein, PrP, and the islet amyloid polypeptide, have been shown to display antimicrobial activity in vitro and ex vivo^31–38^ and induce innate and adaptive immunity^39–43^, with β-amyloid and α-synuclein also shown to protect against various infections in in vivo models^37,38^. Amyloids have also been demonstrated to enable the formation of transmembrane pores in both anionic and zwitterionic lipid membranes, in a mechanism similar to that utilized by antimicrobial peptides for bacterial membrane permeation, suggesting that common principles govern their folding and functions^23,44–47^.

In spite of the recent emerging interest in the correlation between self-assembly and antimicrobial activity, most studies so far have been limited to naturally occurring and intricate antimicrobial peptides and amyloid-forming peptides and proteins, or their relatively complex mimetics, rather than small organic building blocks. As a result, there is a lack of mechanistic understanding of the antibacterial activity of peptide self-assemblies. Thus, there is a genuine need for simpler systems to systematically elucidate the structure–function relationship using minimalistic models that lack the complexities of structure and interpretation of the full-length proteins and peptides, which could be extensively manipulated. An important step in this direction was made by Ghadiri and coworkers in their design of cyclic hexa- and octa-d,l-alpha peptides, which assembled into tubular structures mimicking the facially amphipathic nature of natural antimicrobial peptides and displayed antibacterial activity^48^.

Here we describe the development and comprehensive characterization of the most minimalistic model of self-assembling peptide-based antimicrobial nanostructures reported to date. Interestingly, while classical antimicrobial peptides are usually cationic and amphipathic, the nanostructures assembled by diphenylalanine, which is both neutral and exclusively aromatic, were identified as a minimal self-assembling model, in terms of amino acid content, with notable antibacterial activity. Given the differences between canonical antimicrobial peptides and the diphenylalanine nanostructures, a comprehensive inquiry into the mechanism of action of the later agents was carried out, providing important insights into the significance of the interplay between self-assembly and antimicrobial activity. Furthermore, the minimal nature of this model makes it an attractive platform for the development of alternative antimicrobial therapeutics due to the extensive possibilities for its chemical modifications and allows for further examination of fundamental questions concerning the mechanism of action of self-assembling antimicrobial peptides.

## Results

### Inhibition of bacterial growth

In order to identify the shortest possible peptide that can facilitate antimicrobial activity by molecular self-assembly, we applied a minimalistic approach using diphenylalanine, the core recognition module of the β-amyloid polypeptide^49^, and one of the most prominently studied self-assembling and nanostructure forming bioorganic building blocks^50^. In order to gain a better understanding of the significance of self-assembly to antibacterial activity, assembled diphenylalanine (FF), as well as diglycine (GG), a dipeptide that does not self-assemble, and non-assembled diphenylalanine, which consists of diphenylalanine at sub-critical concentrations (bellow 0.76 mg/ml^51^), were examined. *E. coli* was chosen as a model organism for the analysis, as alongside the well-established laboratory protocols for its handling, it is the pathogenic agent in some of the most common worldwide infections and has developed widespread resistance to last-line classical antibiotics. Furthermore, the transcriptional response to antimicrobial peptide treatment and membrane disruption is well known for this organism^52,53^, allowing us to probe the mechanism of action of the studied compounds and to compare it to that of known antimicrobial peptides. The strain utilized in this study is *E. coli* ATCC 25922, a clinical isolate widely used for testing the susceptibility of bacteria to antibiotics and antimicrobial peptides. Although not considered a pathogenic strain, it is a widely acceptable model for the pathogenic *E. coli* O-strain human pathogens.

**Fig. 1:**
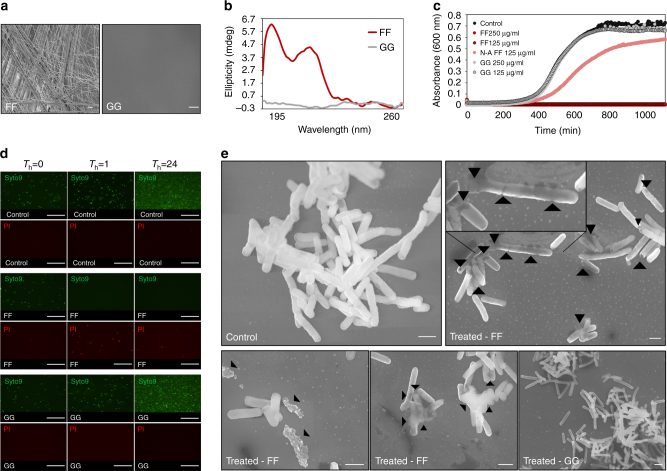
The diphenylalanine nanostructures hinder *E. coli* growth and viability and severely damage bacterial morphology. **a** Morphology of the nanostructures formed by the studied peptides. Micrographs were obtained via scanning electron microscopy. Scale bar is 10 µm. **b** Secondary structure of the studied peptides as obtained by circular dichroism (CD). CD spectra were obtained for both of the peptides diluted in ultra-pure water at 25 °C. **c** Kinetics of the inhibition of bacterial growth. The effect of the diphenylalanine nanostructures and controls on bacterial growth was evaluated by turbidity analysis via absorbance readings at 600 nm of bacteria treated overnight. **d** Effect of the diphenylalanine nanostructures on bacterial viability. Bacterial viability was evaluated using the Live/Dead backlight bacterial viability kit. Green fluorescence of the Syto9 probe indicates bacterial cells with an intact membrane, while red fluorescence of Propidium Iodide (PI) indicates dead bacterial cells. Scale bar is 50 µm. **e** Evaluation of the effect of the diphenylalanine nanostructures on bacterial morphology. Micrographs were obtained using a high-resolution scanning electron microscope. Scale bar is 1 µm

Lyophilized powders of each peptide were heat treated to allow them to reach their monomeric state and were then cooled down gradually to room temperature overnight. Biophysical analysis was carried out using circular dichroism (CD) spectroscopy and scanning electron microscopy (SEM). The formation of unbranched nanotubes was observed for FF, whereas GG did not form structures of any kind and did not present a CD signal correlated with secondary structure formation (Fig. 1a, b).

In order to evaluate their antibacterial effects, the peptide samples were added to an *E. coli* culture, and the antimicrobial activity of each compound was evaluated via minimum inhibitory concentration (MIC) analysis, as well as by kinetic growth inhibition analysis (Fig. 1c; Supplementary Fig. 1). FF exhibited significant activity toward *E. coli*, as overnight incubation at 125 µg/ml completely inhibited bacterial growth, with a reduction of 7.1 log (10) CFU/ml (Fig. 1c; Supplementary Fig. 1). In contrast, GG did not affect bacterial growth at this concentration, nor at any concentration up to 250 µg/ml (Fig. 1c), and the non-assembled diphenylalanine samples inhibited bacterial growth by 15–20% (Fig. 1c). Furthermore, in order to assess the spectrum of the antimicrobial activity of FF, MIC analyses were carried on an additional Gram-negative bacterium, *R. radiobacter*, and two Gram-positive bacteria, *S. epidermidis* and *L. monocytogenes*. The MIC of FF was found to be 250 µg/ml for *R. radiobacter* and *S. epidermidis* and 125 µg/ml for *L. monocytogenes* (Supplementary Table 1). At these concentrations, there was a 7.3, 6.5, and 7.1 log reduction of bacteria, respectively (Supplementary Table 1). Overall, the activity of the FF nanostructures appears to be broad-spectrum in nature. To directly assess bacterial viability, the treated and control bacteria were subjected to Live/Dead viability analysis at three time points (*T*_h_ = 0, 1, 24) (Fig. 1d). After 1 h of treatment with FF, significant bacterial cell death could be observed and no living bacterial cells were identified. This continued for 24 h, while GG did not affect bacterial viability (Fig. 1d).

### Inducement of significant damage to bacterial morphology

The effect of FF and GG treatment on bacterial morphology was studied following treatment with each of the compounds using electron microscopy. Numerous nicks and tears were evident in the cell membrane of the FF treated bacteria (Fig. 1e). Membrane fusing, clumping, and disintegration were also abundant in these bacteria, which appeared deflated (Fig. 1e). Cellular debris as well as lysis of the cells were also evident (Fig. 1e), while the morphology of bacteria treated with GG resembled that of untreated control bacteria, with no nicks, tears, or membrane disintegration and clumping observed (Fig. 1e).

### Interaction with model membrane systems

**Fig. 2:**
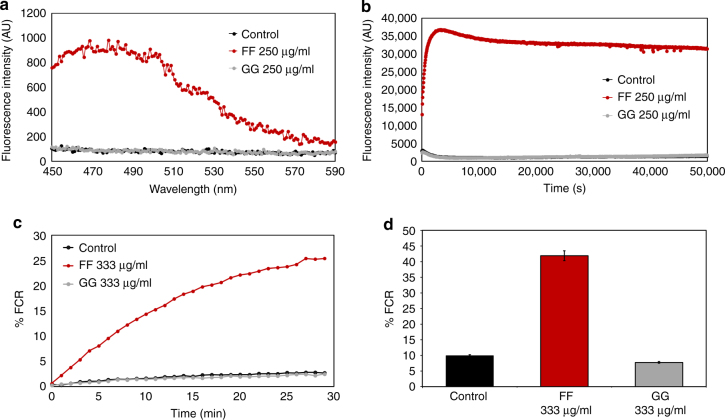
The diphenylalanine nanostructures cause outer and inner membrane perturbations in *E. coli* and interact with model membrane systems. **a** Outer membrane permeation by the diphenylalanine nanostructures. Outer membrane permeation was determined using the ANS sulfonic amine fluorescent probe that displays enhanced fluorescence upon exposure of hydrophobic regions. **b** Cytoplasmic membrane depolarization by the diphenylalanine nanostructures. The cytoplasmic membrane depolarization activities were determined using the membrane potential sensitive dye DiSC3(5). **c**, **d** The diphenylalanine nanostructures interact with model phospholipid membrane systems. Membrane interactions of each compound were determined via measurements of the color transitions induced upon interaction of the compounds with DMPE/DMPG/PDA (1:1:3) vesicles. **c** Kinetics of the initial membrane interaction of the diphenylalanine nanostructures. **d** Interaction of the diphenylalanine nanostructures with the model membrane systems after incubation for 24 h

The abundant perturbations and severe morphology disruption caused by FF treatment point to the bacterial membrane as the main target of the diphenylalanine nanostructures. Therefore, we evaluated their ability to induce membrane permeation and depolarization and directly interact with model membrane systems, subsequently shedding light on their mode of action. Following treatment with FF, significant outer membrane permeation was observed by monitoring the change in the fluorescent properties of the 8-anilino-1-naphthalenesulfonic acid (ANS) dye, a probe which displays increased fluorescence upon binding to hydrophobic membrane regions (Fig. 2a). Furthermore, rapid inner membrane depolarization was observed in samples treated with FF, as indicated by the rapid increase of the fluorescence intensity of the 3,3′-dipropylthiadicarbocyanine iodide (diSC3(5)) lipophilic potentiometric probe (Fig. 2b). Next, we evaluated the propensity of the studied compounds to associate with synthetic phospholipid groups present in bacterial membranes by utilizing a model membrane system^54,55^ comprising of phosphatidylethanolamine (PE) and phosphatidylglycerol (PG) liposomes. The interaction of FF with this system was rapid and continued to grow two-fold over 30 min and four-fold over night (Fig. 2c, d). The kinetics of both outer membrane permeation and inner membrane depolarization by FF, as well as the interaction with model membrane systems, match the kinetics of bacterial cell death observed using the Live/Dead analysis (Fig. 1d), suggesting that the bacterial cell death was associated with permeation and depolarization of the outer and inner membranes, respectively. In contrast, GG did not cause permeation or depolarization of the bacterial membrane and did not display any interaction with the model membrane system (Fig. 2).

### Upregulation of stress response-associated genes

**Fig. 3:**
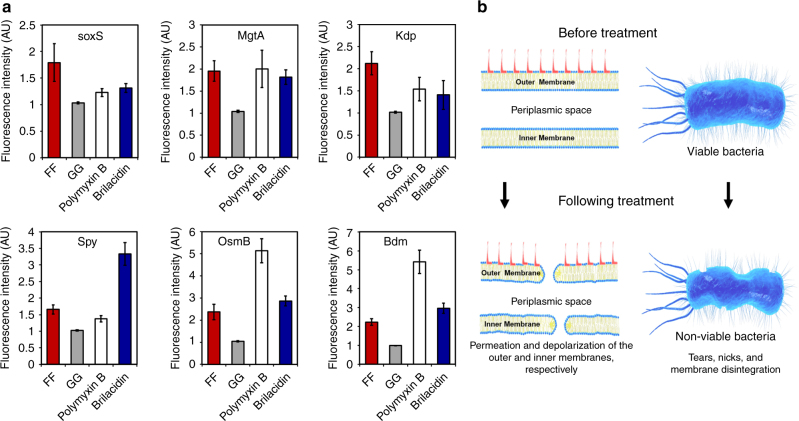
The effect of diphenylalanine nanostructure treatment on the bacterial stress response and their proposed mechanism of action. **a** Treatment with diphenylalanine nanostructures causes upregulation of cellular stress-related genes. *E. coli* bacteria were transformed with plasmids containing each of the six evaluated genes fused to mCherry and the level of fluorescent signal of mCherry, which correlates to the level of expression of the corresponding gene, was measured following the indicated treatments. **b** Model of the proposed mechanism of action of the diphenylalanine nanostructures on Gram-negative bacteria. The interaction of the diphenylalanine nanostructures with the bacterial membrane causes outer membrane permeation and inner membrane depolarization, resulting in severe changes to membrane morphology, such as the appearance of nicks and tears, leading to bacterial growth inhibition and cell death

In order to gain further insight into the mechanism of action of the diphenylalanine nanostructures, we evaluated the response of bacteria to sub-lethal concentrations of these agents as this type of analysis has been shown to be beneficial to the understanding of the mechanism by which bacteria perceive and react to physiological perturbations caused by antibacterial treatment^52,53^. We evaluated the effect of FF treatment on the activation of bacterial responses implicated in sensing cell envelope stress, including the *rcsCB*, *phoQP*, *cpxAR*, *baeSR*, and *kdpDE* two-component systems, as well as the *marA*/*soxS* antibiotic-resistance and superoxide stress response regulators. These signal transduction pathways have been previously shown to be transcriptionally upregulated following treatment with membrane-active antibacterial agents, such as arylamide foldamers (antimicrobial peptide mimetics) and polymyxin B (an extensively studied cyclic antimicrobial peptide)^52,53^. Reporter strains were constructed by transforming *E. coli* with plasmids containing a fluorescent mCherry reporter under the control of promoter sequences of each of the evaluated genes: *sox*S (regulated by marA/soxS), *mgt*A (regulated by *pho*QP), *kdp* (regulated by *Kdp*DE), *spy* (regulated by *cpxAR and baeSR*), and *osm*B and *bdm* (both regulated by *rcs*CB). The bacteria were treated with FF, GG, and controls, as well as with brilacidin (an arylamide foldamer) and polymyxin B, and the fluorescent signal was measured over time to assay the upregulation of each stress response pathway. The signal detected following FF treatment increased over 1.6-fold for each of the studied genes, while GG did not trigger overexpression of these genes (Fig. 3a). The most significant upregulation caused by FF (2.3-fold) was observed for *osm*B and *bdm*, two genes that are part of the *rcs*CB system, which has been previously shown to be induced by treatments that cause outer-membrane perturbation and by changes in osmoregulation^56^. In most cases, the response to FF was comparable to that of brilacidin and polymyxin B, much larger and more complex antibacterial agents, that are both known to strongly interact with Gram-negative bacterial membranes^52,53^. Taken together, these results point to the bacterial membrane as the main target of FF and support our findings that FF causes membrane permeation and depolarization as indicated by the diSC3(5) and ANS assays, as well as the interaction of FF with model membrane systems and our observation of the severe effect of FF treatment on bacterial morphology.

### Biocompatibility and specificity

**Fig. 4:**
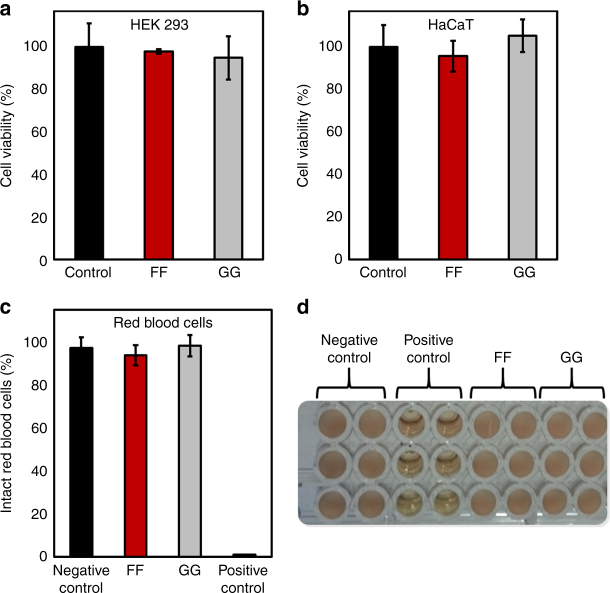
The diphenylalanine nanostructures are non-hemolytic and non-cytotoxic toward human cell lines. **a**, **b** MTT cell viability analysis. The cytotoxicity of the diphenylalanine nanostructures toward two human cell lines was evaluated by the MTT assay using the NAD(P)H-dependent Tetrazolium dye following over-night treatment with the indicated compounds. **a** HEK 293 embryonic kidney cell line. **b** HaCaT keratinocyte cell line. **c**, **d** Hemolysis analysis. The hemolytic activity of the diphenylalanine nanostructures was assessed by incubating defibrinated sheep blood with the compounds and calculation of the subsequent hemolysis from absorbance readings at 451 and 405 nm. The percentage of non-disrupted blood cells is presented **c**, as well as the treated pelleted erythrocytes **d**

Next, the biocompatibility and specificity of the observed membrane interactions to bacterial cell membranes was examined by evaluation of the cytotoxicity of the diphenylalanine nanostructures towards human cell lines, as well as their hemolytic activity. Following treatment with the studied compounds, the viability of both the HEK293 kidney cell line and the HaCaT keratinocyte cell line was assessed via the MTT based cell viability assay. All compounds exhibited remarkable biocompatibility, as >95% of the cells were viable following treatment with 250 µg/ml of FF, a concentration twice as high as the minimum inhibitory concentration against *E. coli* (Fig. 4a, b). Hemolytic activity was evaluated by spectroscopic measurements of the amount of hemoglobin released from red blood cells following treatment with FF and controls. After incubation with 250 µg/ml of FF, over 98% of red blood cells remained intact and undisrupted (Fig. 4c, d). These results demonstrate that the FF toxicity is not a general phenomenon, but rather specific, toward prokaryotic cells.

### Conference of antibacterial activity to tissue scaffolds

**Fig. 5:**
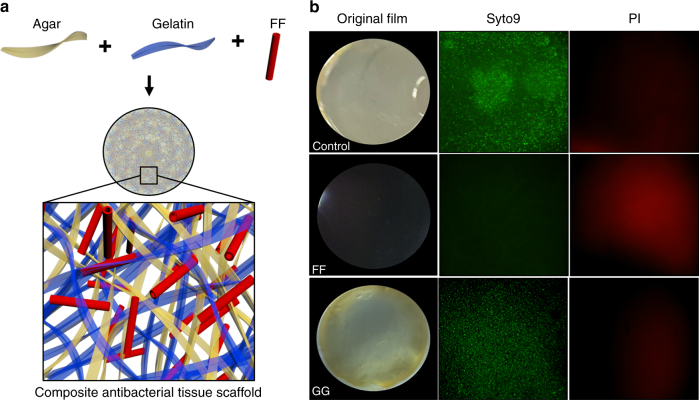
Development of diphenylalanine nanostructure incorporated composite antibacterial tissue scaffolds. **a** Model of the composite diphenylalanine nanostructure incorporated antibacterial tissue scaffolds. **b** Incorporation of diphenylalanine nanostructures into the agar-gelatin tissue scaffold completely hinders growth of bacteria on the scaffold. Bacterial growth was evaluated by turbidity and by Live/Dead bacterial viability analyses

To examine the potential use of the diphenylalanine nanostructures as antimicrobial conferring agents in tissue scaffolds, FF was incorporated into agar-gelatin films, which have been previously shown to be safe to eukaryotic cells and do not possess inherent antimicrobial properties (Fig. 5a). Following synthesis and incorporation of the nanostructures into the matrices, we assessed the antibacterial activity of these hybrid films. As verified by both light microscopy and Live/Dead analysis, the films infused with FF exhibited complete inhibition of bacterial growth as compared to the control films, while normal bacterial growth was observed on GG-infused films (Fig. 5b). The successful generation of tissue scaffolds with intrinsic antibacterial capabilities by incorporation of these antibacterial nanostructures into biocompatible matrices provides the basis for an alternative approach to the design and development of enhanced antibacterial biomaterials.

## Discussion

The pioneering work of Kumar et al.^37^ demonstrated the in vivo protective activity of the 42 amino-acid long β-amyloid polypeptide against potentially lethal infections. While it provided very important insights on the potential physiological role of amyloids, it is difficult to discern the physicochemical attributes that govern the antibacterial activity of this amyloidogenic peptide due to its length and complexity. Thus, we turned to diphenylalanine, the central recognition module of β-amyloid that was identified as a fundamental self-assembly motif, in order to gain insights into the significance of the interplay between self-assembly and antimicrobial activity.

The results of this study demonstrate that the interaction of diphenylalanine with bacterial membranes results in membrane permeation and depolarization, induces upregulation of stress response regulons and causes severe damage to bacterial morphology, thereby inhibiting bacterial growth and inflicting bacterial cell death (Fig. 3b). Furthermore, the incorporation of diphenylalanine into tissue scaffolds introduces antimicrobial capabilities to these matrices. Interestingly, diglycine was devoid of any of these activities while non-assembled diphenylalanine samples, which consisted of the peptide at sub-critical concentrations, inhibited bacterial growth by 15–20%. These results thus underline the significance of self-assembly to the antimicrobial activity and membrane interacting capabilities of diphenylalanine and demonstrate that the antibacterial activity of diphenylalanine cannot be attributed merely to chaperon-like or other non-specific interactions.

The minimal nature of the diphenylalanine motif makes this chemical entity both the shortest antimicrobial peptide-based agent reported thus far and an important platform for the development of alternative antimicrobial agents and materials, due to the extensive possibilities for its chemical modifications, its low cost and high purity. Such modifications may include the incorporation of basic amino acids, non-natural amino acids, and additional functional moieties, which have been shown to greatly improve the potency of short self-assembling antimicrobial peptide-based agents^57–60^. As the MIC of this building block is currently relatively high, these modifications should allow for the development of more potent diphenylalanine-based antimicrobial agents. Furthermore, the biophysical and biochemical characteristic of this building block are also attractive in terms of the potentially low probability for development of bacterial resistance. While several bacterial strains have developed countermeasures to limit the efficacy of classical and synthetic antimicrobial peptides, mainly by electrostatic repulsion and reduced binding following modification of cell envelope molecules, the hydrophobic and non-cationic nature of diphenylalanine would thus make the acquisition of resistance by such membrane modifications challenging. Taken together, these attributes and the results presented in this study ratify the significance of diphenylalanine as a minimal self-assembling antimicrobial building block, and serve to instruct the development of compelling antimicrobial agents and biomedical materials. Furthermore, this work expands on the growing body of evidence pointing to a functional link between self-assembly and antimicrobial activity and provides an important platform for the further investigation of this interplay as well as design principals towards an alternative approach to the development of antimicrobial drugs and biomaterials.

## Methods

### Sample preparation

Lyophilized powders of each of the peptides (Bachem, Switzerland) were dissolved in ultra-pure water (BioInd, Israel) to a concentration of 1 mg/ml and heated for 50 min at 90 °C, allowing for the peptides to reach the monomeric state, and were then allowed to cool gradually to 25 °C over-night. This treatment allowed for the self-assembly and nanostructure formation of FF but not for GG. Non-assembled diphenylalanine samples were prepared by dissolving lyophilized powder of all-l diphenylalnine in ultra-pure water to a concentration of 0.5 mg/ml and heating for 50 min at 90 °C. This concentration is beneath the critical concentration of diphenylalanine self-assembly in water, thus restricting self-assembly and nanostructure formation.

### High-resolution scanning electron microscopy for peptide samples

Following nanostructure formation treatment 10 µl of each compound was deposited on a glass coverslip and dried under ambient conditions overnight. Samples were then coated with Cr. Micrographs were recorded using a JSM-6700F FE-SEM (JEOL, Tokyo, Japan) operating at 5 KV.

### High-resolution scanning electron microscopy for bacterial samples

Bacterial samples were centrifuged at 5000 RPM for 5 min, washed thrice in PBS and fixed in 2.5% glutaraldehyde in PBS buffer for 1 h. Samples were then washed thrice in PBS and fixed in 1% OsO_4_ in PBS buffer for 1 h, followed by a dehydration series with ethanol. Samples were then left in absolute ethanol for 30 min and placed onto glass coverslips, followed by critical point drying and coating with gold. Micrographs were recorded using a JEOL JSM-6700F FE-SEM scanning electron microscope operating at 10 KV. Micrographs displayed are representative of three independent experiments conducted.

### High-resolution transmitting electron microscopy for peptide samples

FF nanostructures which were formed at either 1 mg/ml (above their critical concentration) or 0.5 mg/ml (under their critical concentration) were both diluted to 0.125 mg/ml and 10 µl of each sample was immediately subjected to high-resolution transmitting electron microscopy utilizing a Tecnai 12 TWIN TEM (FEI) transmitting electron microscope. Micrographs were obtained for three separate repetitions of each dilution and 50 nanostructures were measured for both their width and length (a total of 150 nanostructures were measured). The results of this analysis can be found in Supplementary Fig. 2.

### CD spectroscopy

CD spectra were collected with a Chirascan spectrometer (Applied Photophysics, Leatherhead, UK), which was fitted with a Peltier temperature controller fixed at 25 °C, using a rectangular quartz cuvette with an optical path length of 0.1 mm (Hellma Analytics, Germany). Following nanostructure formation treatment, samples were diluted to 0.04 mg/ml and data acquisition was performed in 1 nm increments at a range of 190–260 nm with a spectral bandwidth of 1.0 nm and 3 s integration time, at the linear range (<1.5) of absorbance. The spectrum of each sample was collected thrice and averaged. Spectra were baseline corrected with ultra-pure water (BioInd, Israel) which was collected similarly. Data processing was done using Pro-Data Viewer software (Applied Photophysics, Leatherhead, UK).

### MIC analysis and kinetic growth inhibition analysis

MICs were determined using the microdilution assay. Following nanostructure formation treatment all test compounds were diluted by serial two-fold dilutions in M9 minimal media in Corning® (3879) 96-well plates (Sigma-Aldrich, Israel). *E. coli* bacteria (ATCC 25922) were grown overnight in M9 minimal media and diluted 1000-fold in M9 and grown for 5 h at 37 °C. In Corning® (3596) 96-well plates, 75 µl of the serial two-fold dilutions of each test compound were added to 75 µl of growth medium containing bacteria (5 × 10^6^ CFU/ml). Bacteria and test compounds were incubated overnight at 37 °C and kinetic growth inhibition was determined by optical density measurements (600 nm) using a Biotek Synergy HT microplate reader. Evaluation of the reduction in CFUs was obtained by plating bacterial samples before and after overnight treatment on LB-agar plates. Colony-forming units (CFUs) were counted after overnight incubation at 37 °C. The MIC was considered the lowest peptide concentration that showed no increase in optical density and no CFU growth overnight. Kinetic analysis and CFU reduction displayed are representative of three independent experiments conducted.

### Live/Dead staining for the evaluation of the effect of compounds on bacterial growth

*E. coli* bacteria (ATCC 25922) were grown overnight in M9 minimal media and diluted 1000-fold in M9 and grown for 5 h at 37 °C. Following nanostructure formation treatment 500 µl of each test compound diluted to 0.5 mg/ml in M9 minimal media was added to 500 µl of growth medium containing the bacteria (5 × 10^6^ CFU/ml). At each time point (initial incubation, 1 and 24 h) samples were taken and washed with saline, incubated for 15 min in a solution containing propidium iodide and Syto9 (L13152 LIVE/DEAD^®^ BacLight™ Bacterial Viability Kit, Molecular Probes, OR, USA) and washed with saline again. Fluorescence emission was detected using an ECLIPSE E600 fluorescent microscope (Nikon, Japan).

### Antimicrobial effect of tissue scaffolds

Following incubation of the scaffolds with bacteria overnight, scaffolds were washed with saline, incubated for 15 min in a solution containing propidium iodide and Syto9 (L13152 LIVE/DEAD^®^ BacLight™ Bacterial Viability Kit, Molecular Probes, OR, USA) and washed with saline again. Fluorescence emission was detected using an ECLIPSE E600 fluorescent microscope (Nikon, Japan). Results displayed are representative of three independent experiments conducted.

### Outer membrane permeability assay

Measurements were performed using the 8-anilinonapthalene-1-sulfonic acid (ANS) uptake assay. *E. coli* bacteria (ATCC 25922) were grown overnight in M9 minimal media and diluted 1000-fold in M9 and grown for 5 h at 37 °C until an OD_600_ of 0.5. Bacteria were then washed with 10 mM Tris HCl, 150 mM NaCl buffer (pH 7.4) and resuspended in the same buffer with 10 µM ANS at an OD_600_ of 0.05. The resuspended bacterial samples were then equilibrated in black Greiner CELLSTAR^®^ (655090) 96-well plates for 20 min. Following nanostructure formation treatment test compounds were then added to the equilibrated bacterial solutions to a final concentration of 250 µg/ml and the changes in the fluorescence emission was measured between 450–600 nm with excitation at 380 nm, using a CLARIOstar® BMG LABTECH microplate reader. 1% TritonX-100 was used as a positive control. Results displayed are representative of three independent experiments conducted.

### Inner membrane depolarization assay

Measurements were performed with the lipophilic potentiometric dye 3,3′-dipropylthiadicarbocyanine iodide (diSC3(5)). *E. coli* bacteria (ATCC 25922) were grown overnight in M9 minimal media and diluted 1000-fold in M9 and grown for 5 h at 37 °C until an OD_600_ of 0.5. Bacteria were then washed with 5 mM sodium HEPES, 2 mM EDTA buffer (pH 8) and resuspended in the same buffer at an OD_600_ of 0.05. The bacterial samples were then incubated with 0.4 µM diSC3(5) in black Greiner CELLSTAR^®^ (655090) 96-well plates for 60–90 min, until a stable reduction of fluorescence was achieved. Following nanostructure formation treatment test compounds were then added to the stabilized bacterial solutions to a final concentration of 250 µg/ml and the changes in the fluorescence signal was monitored continuously using a CLARIOstar® BMG LABTECH microplate reader (excitation, 622 nm; emission, 670 nm). 1% TritonX-100 was used as a positive control. Results displayed are representative of three independent experiments conducted.

### Preparation of vesicles for model membrane system interaction analysis

Vesicles comprised of DMPE and DMPG lipid components as well as the diacetylene monomer 10,12-tricosadiynoic acid (TRCDA) at a ratio of 1:1:3 (DMPE/DMPG/TRCDA) were dissolved in a 1:1 chloroform/ethanol mixture and were dried together in vacuo to constant weight. Deionized water was added to a final concentration of 1 mM, and the samples were then sonicated at 40 W at 70 °C for 3 min using a probe sonicator and subsequently cooled to room temperature. The vesicle solution was stored at 4 °C overnight and then irradiated at 254 nm for 30 s, which resulted in the appearance of an intense blue color, due to polymerization of the diacethylene units.

### Fluorescence spectroscopy for model membrane system interaction analysis

Samples were prepared by adding 30 μl of each of the test and control compounds at 1 mg/ml to 30 μl of the vesicles followed by addition of 30 μl 50 mM Tris-base buffer (pH 8.0). Fluorescence measurements (excitation, 485 nm; emission, 555 nm) were carried out on a Fluscan Ascent microplate reader at a constant temperature of 27 °C, using LP filters with normal slits. The background fluorescence of the vesicles alone was negligible. The fluorescent chromatic response (FCR) was calculated according to the following formula: %FCR = [EmI/Emred]×100%. EmI is the value obtained for the vesicle treated with the test compounds, and Emred is the value obtained for the vesicles treated with the positive control (NaOH 1 M). Results displayed are representative of three independent experiments conducted.

### CD spectroscopy for model membrane system interaction analysis

Following 15-h incubation of the vesicles with the FF samples were diluted 10-fold and CD spectra were collected with a Chirascan spectrometer (Applied Photophysics, Leatherhead, UK) as described in the above CD spectroscopy section. The results of this analysis can be found in the Supplementary Fig. 3.

### Evaluation of the upregulation of bacterial stress response genes

Bacterial cell reporters were used to quantify activation of bacterial stress-response implicated regulons and two-component systems. Plasmids were constructed containing a fluorescent mCherry reporter under the control of the promotor sequences for genes regulated by the following bacterial stress response implicated two-component systems and regulons: *sox*S (regulated by marA/soxS), *mgt*A (regulated by *pho*QP), *kdp* (regulated by *Kdp*DE), *spy* (regulated by *cpxAR and baeSR*) and *osm*B and *bdm* (both regulated by *rcs*CB). Sequences of the plasmid and reporters used can be found in the Supplementary Methods. Plasmids were transformed into *E. coli* bacteria (ATCC 25922) by electro-transformation. Briefly, 50 μl of ~10^9^ electro-competent cells were placed in 1 mm gap cuvettes with ~15 ng plasmid, and transformed with standard settings (1.8 kV, 25 uF, 200 Ohm). The cells were recovered with 1 ml LB and incubated at 37 °C for 1 h with shaking. Transformants were obtained by plating 50 μl of the bacterial solution onto LB Agar petri dishes supplemented with 100 μg/ml ampicillin, and picking colonies following overnight incubation. Transformants were then grown overnight in M9 minimal media supplemented with trace metals and then diluted 1000-fold and grown to mid log phase. The bacterial cultures were then diluted to a final OD_600_ of 0.1 and treated with Polymyxin B at a final concentration of 0.6 µg/ml, Brilacidin at a final concentration of 1.1 µg/ml and test compounds following nanostructure formation treatment at a final concentration of 250 µg/ml, for 7 h. This was followed by measurement of the absorbance of each sample at 600 nm as well as the reading of mCherry fluorescence at 620 nm (excitation at 590 nm). The results presented for each gene were calculated by normalizing the fluorescent signal to OD_600_ for each sample. The results presented are the mean of three independent experiments conducted ±the standard error of the mean.

### Cell cytotoxicity experiments

Test compounds and the negative control (PBS) were dissolved in PBS at 1 mg/ml and heated for 50 min at 90 °C, followed by gradual cooling of the solution overnight. Samples were then diluted two fold in DMEM/Nutrient Mixture F12 (Ham’s) (1:1) (Biological Industries) lacking fetal bovine serum. 1% TritonX-100 in PBS was used as a positive control. 2 × 10^5^ cells/ml of the HaCat and HEK 293 cell lines were cultured in 96-well tissue microplates (100 µl per well) and were allowed to adhere overnight at 37 °C. Cells were plated on only half of the microplate with the other half consisting of growth medium with and without each of the test compounds. Cell viability was evaluated using the 3-(4,5-dimethylthiazolyl-2)-2,5-diphenyltetrazolium bromide (MTT) assay following incubation with the test and control compounds overnight at 37 °C. Briefly, 10 µl of 5 mg/ml MTT dissolved in PBS was added to each well and incubated for 4 h at 37 °C. This was followed by the addition of 100 µl of the extraction buffer [20% SDS dissolved in a solution of 50% dimethylformamide and 50% DDW (pH 4.7)] to each well, and incubation at 37 °C for 30 min. The resulting color intensity was then measured using a microplate reader at 570 nm. The results presented are the mean of three independent experiments conducted ± the standard error of the mean.

### Measurement of hemolytic activity

The hemolytic activity of the compounds was measured as the amount of hemoglobin released by the lysis of erythrocytes. Briefly, 1 ml of sheep blood was centrifuged at 1000×*g* for 5 min at 4 °C. The erythrocytes obtained were washed with three times with PBS (pH 7.2) and resuspended in PBS. Samples of 100 µl of the erythrocyte solution were incubated for 2 h in 96-well plates with each of the test compounds following nanostructure formation treatment in PBS at a final concentration of 250 µg/ml. Intact erythrocytes were pelleted by centrifugation at 1000×*g* for 5 min at 4 °C and the supernatant was transferred to a new 96-well plate. The release of hemoglobin was monitored by measuring the absorbance at 451 and 405 nm. The negative control was PBS treated in the same manner as the test compounds and the positive control was 1% TritonX-100. The results presented are the mean of three independent experiments conducted ± the standard error of the mean.

### Preparation of nanostructure incorporated agar-gelatin gel films

A mixture of (2 wt%) Agar, (1 wt%) gelatin, and (4 wt%) LB was taken and dissolved in PBS (pH 7.4). Following nanostructure formation treatment, samples of each compound were added to this homogeneous mixture to achieve a final concentration of 750 µg/ml sample, 1 wt% agar, 0.5 wt% gelatin, and 2 wt% LB. Glutaraldehyde (0.15 wt%) was added to the mixture to achieve cross-linking of the components in solution and 1 ml of the solution was poured into 24-well plates and allowed to rest overnight. The plates were then placed in an incubator heated at 60 °C for 10 h to allow for the formation of the films. To these, 0.1 mM glycine was added to ensure the blocking of the excess glutaraldehyde and the films were washed with excess water and PBS to remove the unreacted glycine and neutralize the surface. The films were then dried and sterilized using UV irradiation.

### Antibacterial activity of agar-gelatin films

*E. coli* bacteria (ATCC 25922) at an OD_600_ of 0.01 were plated onto the films that were allowed to swell in PBS (7.4) and allowed to grow overnight at 37 °C. Bacterial growth was evaluated by turbidity as well Live/Dead bacterial viability analysis. Results displayed are representative of three independent experiments conducted.

### Data availability

The authors declare that all of the data supporting the findings of this study are available within the article and its Supplementary Information files.

## Electronic supplementary material

Supplementary Information
