# Supplementary material for: Self-assembling dipeptide antibacterial nanostructures with membrane disrupting activity
Source: Nat Commun. 2017 Nov 8;8:1365. doi: 10.1038/s41467-017-01447-x (PMC5678095; doi:10.1038/s41467-017-01447-x)
Supplement: Supplementary file 1 — Supplementary Information [file 41467_2017_1447_MOESM1_ESM.pdf]

Supplementary Table 1. Antibacterial effect of FF on Gram-negative and Gram-positive bacteria.

	Gram-negative bacteria		Gram-positive bacteria	
	E. coli	*R. radiobacter*	*L. monocytogenes*	*S. epidermidis*
	**ATCC 25922**	**ATCC 33970**	**BUG 1361**	**ATCC 12228**
**MIC (mg /ml)**	0.125	0.25	0.125	0.25
**Log reduction at MIC**	7.1	7.3	7.1	6.5

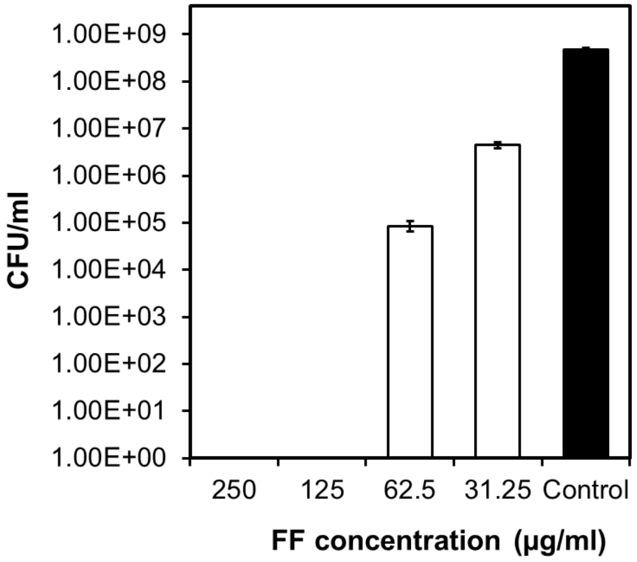

Supplementary Figure 1: Effect of the diphenylalanine nanostructures on the reduction of bacterial colony forming units (CFU) upon treatment. The reduction in CFU analysis was carried out by plating bacteria before and after treatment and comparing the number of CFU.

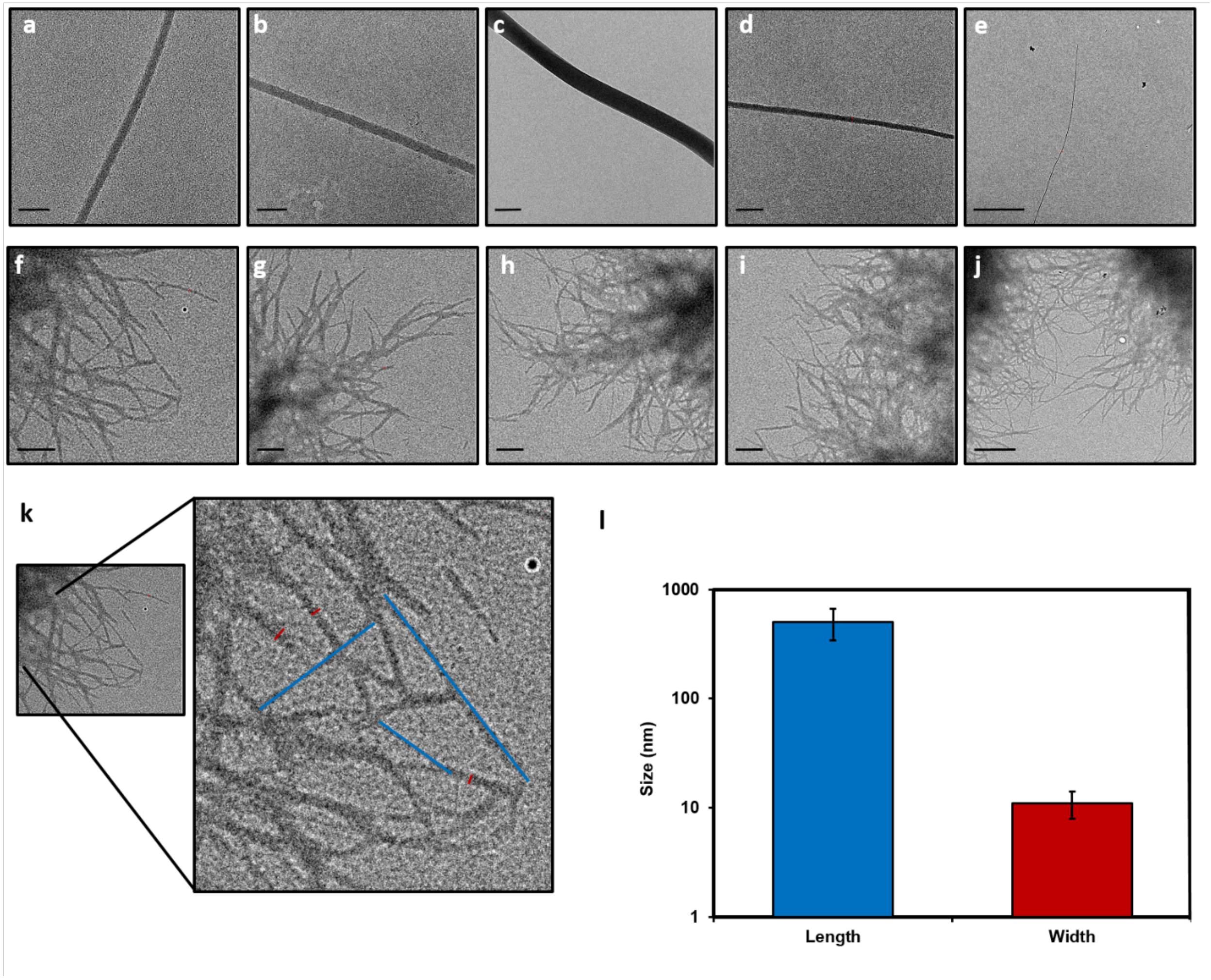

Supplementary Figure 2. Characterization of the width and length of the FF nanostructures. (a-e) Micrographs of the nanostructures observed in the samples of FF initially dissolved to 0.5 mg/ml (under their critical concentration) and then diluted to 0.125 mg/ml. Scale bar is 100nm (a-b), 200nm (c-d) and 2μm (e). **(f-j)** Micrographs of the nanostructures observed in the samples of FF initially dissolved to 1 mg/ml (above their critical concentration) and then diluted to 0.125 mg/ml. Scale bar is 200nm (f-i) and 500nm (j) **(k)** Magnified view of the measured nanostructures. Width was measured along the red lines and length was measured along the blue lines. **(l)** Statistical analysis of the width and length of the nanostructures of the FF samples initially dissolved to 1 mg/ml (above their critical concentration) and then diluted to 0.125 mg/ml. The measured width of the nanostructures was 11nm ± 1.1nm and the measured length was 503nm ± 163nm.

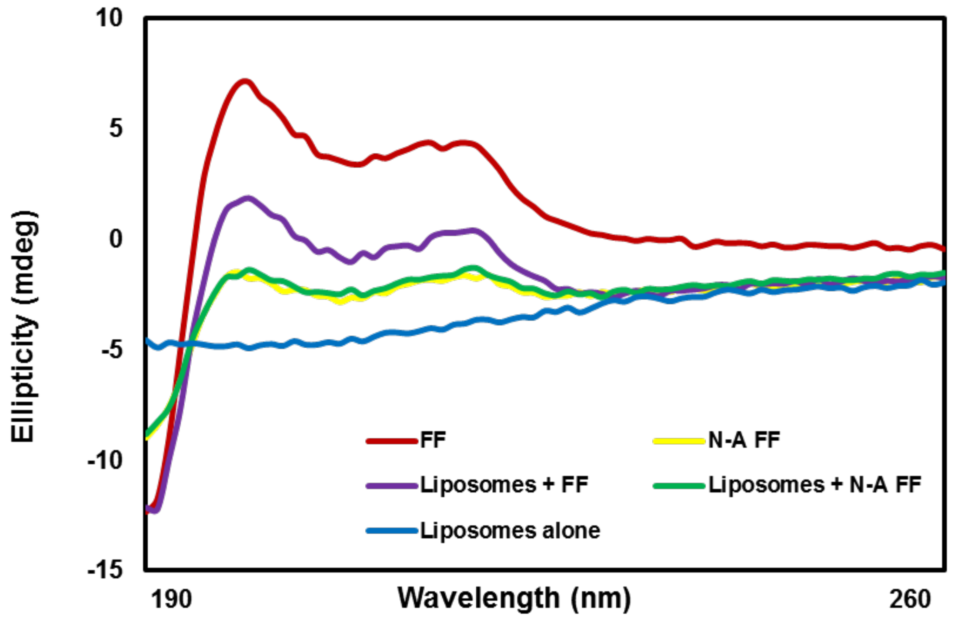

Supplementary Figure 3. CD spectroscopy of the interaction between FF and model phospholipid membrane systems. FF samples dissolved initially to 1 mg/ml (above their critical concentration) or 0.5 mg/ml (under their critical concentration) were incubated with DMPE/DMPG/PDA (1:1:3) vesicles to a final concentration of 0.33 mg/ml or 0.16 mg/ml, respectively. Following 15-hour incubation the CD spectra was obtained for each sample.

Supplementary Methods:

Plasmid and reporters used for the evaluation of the upregulation of bacterial stress response genes. Gibson assembly was used for insertion of the following sequences into the pEXP5-NT plasmid (Invitrogen, ThermoFisher Scientific). Note that the 3' end of each sequence is followed by the ATG of the mCherry reporter.

pKdpA:
CTAAACATTAGTTAAATCATGGCTTTTGCCATTTTTATACTTTTTTTACACCCCGCCC
GCAGATTTTTGCGAAATCTTTGCAGCCAGAATTCTACCCTTCCGGTATCACTTTTAGG
CCACTGGAGGTGCACTGTGAGTGCAGGCGTGATAACCGGCGTATTGCTGGTGTTTTT
ATTACTGGGTTATCTGGTTTATGCCCTGATCAATGCGGAGGCGTTCTGATG

pMgtA:
ACACGCTCGCGGGTGAGCTGGCTCACGCCGCTTTCGTTATTCAGCACCCGGGAAACT
GTAGATTTCCCCACGCCGCTTAAGCGCGCGATATCTTTGATGGTCAGCCGATTTTGC
ATCCTGTTGTCCTGTAACGTGTTGTTTAATTATTTGAGCCTAACGTTACCCGTGCATT
CAGCAATGGGTAAAGTCTGGTTTATCGTTGGTTTAGTTGTCAGCAGGTATTATATCG
CCATAGATGCTACGAATATTATTGGATTCTCCTTATTATTTGCGGCGCTTTTTTCACT
TACCGGAGGTTATATGGAACCTGATCCCACGCCTCTCCCTCGACGGAGATTAAAACT
TTTCCGGTAAGCCCGTCTTTTCACGGCGTTACCGGATGCGTAAGGCCGTGACGTTTT
AACGTCCCTGCTCAGCTTTATTACCTTCAGGTAAGGCTTCGCCACGCCTGAAGACAT
TTCTGTACTGTTTCAGACAGTGCGGAGGGACTCCTTATG

pOsmB:
GATGATTTGCAGTTTGGCAAATCATCCGCTCTAAGATGATTCCTGGTTGATAATTAA
GACTATTTACCTGTTATTAACACTCTCAAGATATAAAATTATTATCAGCGATATAAC
AGGAAGTCATTATCACCTGCGTGATATAACCCTGCGCGCGAGCAGATTTCACGGAAT
AATTTCACCAGACTTATTCTTAGCTATTATAGTTATAGAGAGCTTACTTCCGTGAATC
ATAAATTCAGGAGAGAGTATTATG

pBdm:

GAATATCATGGTGTTAAAATATAAAAATCATCAACCAGGACTAATCTTAACAACGA
AGCGGCAAATATTTGCCATGCTGAATGTGCTTTATAAATCTGCGATCCGTAGCAGAC
ACCATAAATACACAGACACGGAGAATCACTATG

pSoxS:

CTTTTCCATAAATCGCTTTACCTCAAGTTAACTTGAGGAATTATACTCCCCAACAGAT
GAATTAACGAACTGAACACTGAAAAGAGGCAGATTTATG

pSpy:

CATATGGCAAGTGACACGCTGAATTTTATGCCGTTTGAGAAAGGAACATTGCTGGCG
CAGGACGGAGAGGAACGTTTTACCGTAACCCATGATGTAGAGTATGTGTTATTCCCT
AATCCGTTGGTAGCGTTGGGATTACGCGCGGGATTAATGCTCGAAAAAATAAGCTA
ATACTTACCCGCAGAAATCATTCTGCGGGTATTAAGTTGCTTTCTTATAAATTAATAC
AGATTAATCCTGGTTATTTGCTTTATTTATCACCAGTCATCCGGTATAGTTCTTCATA
ATCTCTGCAAAATCATCGTGTTGTAATATTCTCTCATCACTCTCCATCAAATTTTCTTT
TTTTCTCCATAATTGGCGCAAAAGTGTTTTTTACACTTTCATTGTTTTACCGTTGCTCT
GATTAATTGACGCTAAAGTCAGTAAAGTTAATCTCGTCAACACGGCACGCTACTTAA
GAAAGCCGTAATAAATAACTGAAAGGAAGGATATAGAATATG
